# Application of microarray technology in pulmonary diseases

**DOI:** 10.1186/1465-9921-5-26

**Published:** 2004-12-07

**Authors:** Argyris Tzouvelekis, George Patlakas, Demosthenes Bouros

**Affiliations:** 1 Department of Pneumonology, Medical School, Democritus University of Thrace, Greece

**Keywords:** Microarrays, pulmonary fibrosis, asthma, chronic obstructive pulmonary disease, acute lung injury, pulmonary edema, sarcoidosis, pulmonary diseases

## Abstract

Microarrays are a powerful tool that have multiple applications both in clinical and cell biology arenas of common lung diseases. To exemplify how this tool can be useful, in this review, we will provide an overview of the application of microarray technology in research relevant to common lung diseases and present some of the future perspectives.

## Introduction

**Figure 1:**
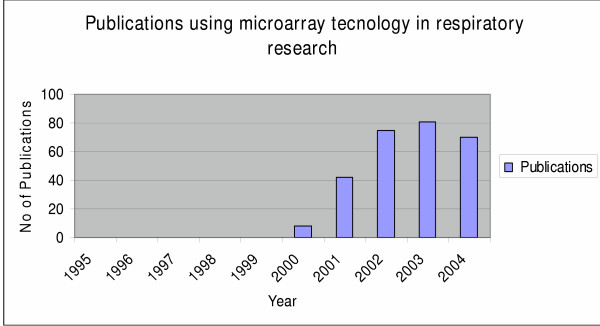
Diagram showing the number of published papers using microarray technology in respiratory research the last ten years since 1995 when microarrays were first applied in clinical medicine.

**Figure 2:**
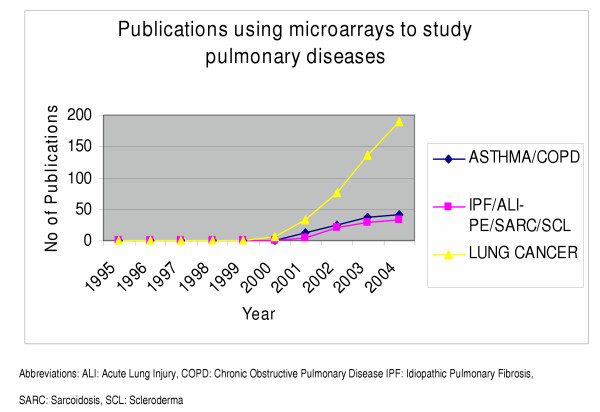
Diagram showing the number of published papers in research relevant to common lung diseases such as asthma, COPD, IPF, ALI/PE, SARC, SCL, lung cancer, the last ten years since 1995 when microarrays were first applied in clinical medicine.

Microarray technology is rapidly becoming a standard technology used in research laboratories all across the world. Since its first application in the mid 1990s [1] microarray technology has been successfully applied to almost every aspect of biomedical research [2-7] with over 250 papers in respiratory research alone. Research conducted the last ten years has elevated the status of microarray technology from poorly understood and doubtfully applied in the fields of medicine to one that requires attention when the examination of clusters of genes in a single experiment is considered. Far more progress has been made toward an understanding of the pivotal role of microarrays in respiratory research by providing the scientists well-established knowledge concerning numerous genes that can be used as potential drug targets, mediators and inflammatory molecules with important cellular functions, evidence that captured the interest of both clinicians and researchers and caused a consecutive year by year rise of the applications of microarrays in experiments designed to study pulmonary diseases. Thus microarray technology since its first application [8] in the field of respiratory medicine has already been used the past five years in almost every aspect of respiratory research with an increased rate of application resulting to an overall of approximately 250 published papers until now (Figures 1, 2). Though the majority of experiments using microarray platforms was designed to study lung cancer (Figure 2) we excluded from this review this data, because considering the number of published papers, the enormous data derived from these experiments could compile a separate review article on its own. The scope of this review was based on the fact that although there are numerous original published papers using this pioneering method, the number of review articles summarizing the importance of microarrays in the research field relevant with pulmonary diseases still remains inadequately small.

### Applications of microarrays in medicine

DNA microarrays, microscopic arrays of large sets of DNA sequences immobilized on solid substrates, are valuable tools in areas of research that require the identification or quantitation of many specific DNA sequences in complex nucleic acid samples [8]. They are ordered samples of DNA and each sample represents a particular gene. These arrays can then be assayed for changes in the expression patterns of the representative genes after different treatments, different conditions or tissue sources. There are numerous ways to measure gene expression including northern blotting, differential display, serial analysis of gene expression and dot-blot analysis. The problem with all these techniques is that they are unsuitable for the parallel testing of multiple genes' expression. Microarrays, based on Southern's method of nucleotide hybridization, contain multiple DNA sequences (probes) spotted or synthetized on a relatively small surface. This feature of microarrays allows the simultaneous monitoring of the expression of thousands of genes, thus providing a functional aspect to sequence information, in a given sample [9]. Currently, genomic microarrays are used in medicine for the following purposes: [10-13]

1. Determination of transcriptional programs of cells for a given cellular function (e.g., cell function, cell differentiation, etc.) or when they are exposed to certain conditions leading to activation, inhibition or apoptosis.

2. Compare and contrast transcriptional programs to aid diagnosis of diseases, predict therapeutic response and provide class discovery and sub-classification of diseases.

3. Identification of genome-wide binding sites for transcriptional factors that regulate the transcription of genes.

4. Prediction of gene function.

5. Identification of new therapeutic targets (target identification, target validation, and drug toxicity).

6. Development of public databases that will help us understand the functioning of complex biological systems.

7. Genetics of gene expression: Although this is a relatively new study field, it is advancing rapidly with major implications in complex clinical traits by the identification of promising candidate genes. Thus, we briefly review the current implementations of this novel approach highlighting its necessity in the research field. Treating mRNA transcript abundances as quantitative traits and mapping gene expression quantitative trait loci for these traits has been pursued in gene-specific ways. Unlike classical quantitative traits, the genetic linkages associated with transcript abundance permits a more precise look at cellular biochemical processes. Schadt et al. [14] described comprehensive genetic screens of three specific transcriptomes by considering gene expression values as quantitative traits. Authors treated the gene expression levels derived by a microarray analysis in mice liver tissues as quantitative traits in a standard linkage analysis using evenly spaced autosomal markers. Interestingly they found that a substantial portion of these genes had at least one significant gene expression quantitative trait locus (eQTL) depending on the LOD (log odds ratios) scores. Since transcript abundances are increasingly used as surrogates for clinical traits, knowledge about their genetic control can help dissect the genetics of complex traits. In the same study investigators revealed the importance of LOD scores to differentiate whether the expression levels of the genes under study is regulated by variations within the gene itself (cis) or at a separate locus (trans). They found that eQTL with LOD scores are cis acting (gene affects transcription of the gene itself) in most cases, whereas moderately significant eQTL are trans acting (genes acting on the transcription of other genes). Furthermore this study undertook an investigation on how the heritability of gene expression can be studied within and between families and demonstrated that a significant portion of differentially expressed genes derived from reference families had a detectable genetic component. The latter finding suggests that this group of genes may serve as novel therapeutic targets for complex human diseases, given that their degree of genetic control was so readily identifiable in a small number of families.

### Microarray technologies

DNA microarrays are used to estimate the levels of mRNA in the cell. The process can be described in three steps:

1) *Array construction*: Currently, there are two widely used microarray technologies:

• In situ synthetized oligonucleotide (20–25 mers) microarrays-mainly oligonucleotides synthetized by photolithography or ink-jet technology on a glass surface.

• Spotted, in glass or nylon membrane matrices, microarrays-mostly created by robotic printing of pre-prepared cDNAs or oligonucleotides (polymerase chain reaction-PCR-generated products from cDNA libraries or clone collections) [9,15].

2) *Sample preparation and array hybridization*: The next step in the microarray experiment is to prepare the material that will be hybridized with the microarray. Gene expression is measured by the amount of mRNA therefore it must be extracted from the sample cells or tissues. For high-density microarrays one has to convert mRNA into cRNA, whereas for spotted arrays one can use mRNA, cDNA, or cRNA. The RNA needs to be labeled so that the detecting machinery can measure the quantity of RNA present. In oligonucleotide microarrays mRNA is extracted from experimental samples and is labeled with a fluorescent oligonucleotide (biotin). The biotin labeled cRNA and each sample is hybridized to a separate array, the array is scanned and absolute expression levels are obtained for each probe by using a dedicated laser scanner. In contrast, in spotted microarrays, mRNA is extracted from a sample and a control and one is labeled with cy-5 (red fluorescent dye) and the other with cy-3 (green fluorescent dye). Expression values are based on the competitive hybridization of the two samples being directly compared on a single array. Conventionally, in radioactive nylon membrane arrays RNA probes are labeled with P^33 ^or P^32 ^dCTP during a reverse transcription reaction. The great advantage of nylon microarrays is that they require relatively small amounts of PCR products because radioactive targets have a higher intrinsic detectability whereas in glass arrays the quality of RNA is not as critical as it is in nylon arrays. Therefore nylon arrays are mainly used when sample material is scarce or a small number of genes need to be assayed [16,17].

**Figure 3:**
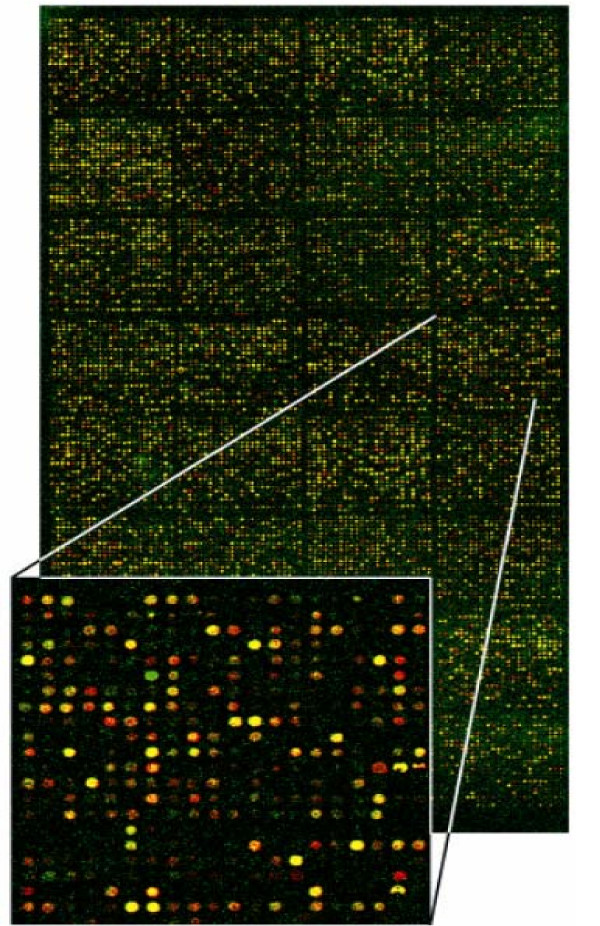
Image from laser scanning confocal microscope of a DNA microarray slide. mRNA, is extracted from a sample and a control and after its transcription into more stable cDNA, one is labeled with cy-5 (red fluorescent dye) and the other with cy-3 (green fluorescent dye). The two cDNA populations are allowed to hybridize to the same microarray slide. If particular mRNA from the sample is in abundance, the spot with a complementary probe will be red (induction of gene expression in sample condition); if the concentration of the particular mRNA is higher in the control, the spot will be green (induction of gene expression in control condition). If both samples contain the same amount of a given mRNA the spot will be yellow (equal gene expression in both conditions). (Adapted with permission of Dr Karameris Andreas.)

*3) Image analysis and data acquisition: *The laser causes excitation of fluorescently or radioactively labeled cDNA probes. Only the spots representing mRNAs in the sample give emission signals. The emission is measured using a scanning confocal laser microscope for fluorescently labeled arrays or a flat-bed scanner for radioactive nylon arrays and finally data is analyzed by appropriate software. In spotted microarrays using fluorescent probes if particular mRNA from the sample is in abundance, the spot with a complementary probe will be red; if the concentration of the particular mRNA is higher in the control, the spot will be green. If both samples contain the same amount of a given mRNA, the spot will be yellow (Figure 3). In nylon membrane microarrays using radioactive probes acquisition of phosphor-representations of radioactive hybridizations is performed with a high resolution digital autoradiography system displaying in real time the quantitative image of radioisotopes deposited on biological samples [18]. In high density oligonucleotide microarrays the absence of the fluorescence of the specific spots means that complementary mRNA is not present in the sample. If the fluorescence is present, the intensity of the signal is a measure of the level of particular mRNAs in the examined cell population [11-19].

### Application of microarrays in pulmonary diseases

#### 1. Microarrays in idiopathic pulmonary fibrosis (Table 1)

**Table 1:** Studies utilizing microarray technology to analyze IPF

Investigator	Microarray type	Species/Sample size	Summary/Key findings	Normalization procedure
	Number of genes	Type of tissue		Replications per data point
Zuo et al.^22^	Oligonucleotide 8.400 genes	5 patients with IPF Lung tissue specimens	Gene expression analysis reveals matrilysin as a key regulator of PF in mice and humans.	Gene expression levels normalized by a scaling factor multiplied to the average of differences of probe pairs (matched-mismatched) / 2 replicates
Kaminski et al.^23^	Oligonucleotide 6.000 genes	30 mice Lung tissue specimens	Global analysis of gene expression in PF reveals distinct programs regulating lung inflammation and fibrosis.	Mean hybridization intensities of all probe sets on each array were scaled to an arbitrary, fixed level/4 replicates
Katsuma et al. ^24^	cDNA 4.224 genes	22 mice Lung tissue samples	Molecular monitoring of bleomycin-induced pulmonary fibrosis by cDNA microarray-based gene expression profiling.	Quantified signal intensities were converted by logarithms of base two 4 replicates
Chambers et al. ^25^	Oligonucleotide 6.000 genes	Human lung fibroblasts	Global expression profiling of fibroblast responses to transforming growth factor-beta1 reveals the induction of ID1.	Gene expression levels normalized by a scaling factor multiplied to the average of differences of probe pairs (matched-mismatched)/ 2 replicates
Liu et al.^26^	cDNA 10.000 genes	12 rats Lung tissue specimens	FIZZ1 stimulation of myofibroblast differentiation.	Average median ratios Cy3/Cy5 values normalized to 1.0/ 4 replicates

Abbreviations: ID: Inhibitor of Differentiation, IPF: Idiopathic Pulmonary Fibrosis, PF: Pulmonary Fibrosis

Idiopathic pulmonary fibrosis (IPF) is a refractory and lethal interstitial lung disease characterized by fibroblast proliferation, extracellular matrix (ECM) deposition and progressive lung scarring. The incidence of IPF is estimated at 15–40 cases per 100.000 per year, and the mean survival from the time of diagnosis is 3–5 yr regardless of treatment [20,21]. The etiology of IPF has remained elusive and the molecular mechanisms are poorly understood. To elucidate the molecular mechanisms that lead to end-stage human pulmonary fibrosis Zuo et al. [22] analyzed lung biopsy samples from five patients with clinically, radiologically and histologically proven pulmonary fibrosis (usual interstitial pneumonia-UIP) and compared to samples from three resected lungs with normal histologic findings, by using oligonucleotide microarrays. Using a combined set of scoring systems they determined that matrilysin (matrix metalloproteinase-MMP-7), a metalloprotease not previously associated with pulmonary fibrosis, was the most informative increased gene in their data set. Immunochemistry demonstrated increased expression of matrilysin protein in fibrotic lungs derived from different patients. Furthermore, in a separate set of experiments matrilysin knockout mice were dramatically protected from pulmonary fibrosis in response to intratracheal bleomycin. Their results identify matrilysin as a mediator of pulmonary fibrosis and a potential therapeutic target. Nevertheless potential limitations of the study include the small sample size and the relative inability of microarray analysis using whole lung homogenates to assess the exact cells that were overexpressing the informative genes. The application of analytic microarray approaches using gene expression signatures of specific cell types coupled with advanced data mining computational tools will ameliorate this hardship.

In another study Kaminski et al. [23] used oligonucleotide microarrays to analyze the gene expression programs that underlie pulmonary fibrosis in response to bleomycin, in two strains of susceptible mice. Microarray analysis performed by different investigators and at different time points demonstrated a considerable overlap between genes induced by bleomycin in these two distinct strains of mice. Differential gene expression in response to bleomycin included upregulation of genes known to be associated with bleomycin-induced lung injury and fibrosis such as transforming growth factor-β1 (TGF-β1), as well as genes not previously associated with the disease. Confirmational studies performed and further verified a portion of the microarray data. Surprising insights were derived from comparing gene expression patterns in response to bleomycin of mice homozygous for a null mutation of the integrin β6 subunit gene (β6 ^-/-^), thus protected from pulmonary fibrosis, and wild type mice. Interestingly a simple hierarchical cluster analysis identified most of the known TGF-β1 inducible genes preferentially induced in wild type mice. The latter finding provides support for the hypothesis that β6 knockout mice are protected from pulmonary fibrosis as a consequence of failure to activate TGF-β. The great importance of this study results from the identification and the global availability of several genes that are likely to be directly relevant to the fibrotic process. However the inability of microarray technology to detect genes that are not included in the array, identify critical proteins that participate in biological responses and ascribe changes in gene expression in specific cellular types limit the scientific rigidity of the data derived and highlights the necessity for combined application of novel approaches.

Furthermore driven by the same perspective idea to investigate the gene expression pattern in bleomycin-induced pulmonary fibrosis, Katsuma et al. [24] constructed a lung chip derived from a normalized lung cDNA. They performed large-scale analyses of gene expression and illuminated a time-dependent change in the expression profile of genes related to the inflammatory and fibrotic responses in this model of pulmonary fibrosis, similar to that observed by Kaminski et al [18]. Using cluster analysis they classified genes into groups based on a time-dependent gene expression. Interestingly this profile was well correlated with observed histopathological changes and data was confirmed with real time-(RT)-PCR methods. Nevertheless apparent inconsistencies with the gene expression pattern revealed by Kaminski et al. [23] highlight the inability of microarray approach in distinguishing changes in transcriptional regulation from changes in cellular composition of the organ being studied. Thus, it is likely these discrepancies between findings of the two studies to represent differences in cellular composition, rather than differences in transcriptional regulation.

One of the most informative studies scrutinizing the global gene expression profile of fibroblasts in response to one of their most potent activators, TGF-β1, has been published by Chambers et al. [25] Gene expression analysis of human lung fibroblasts treated with TGF-β1 has led investigators to uncover novel TGF-β1-inducible genes including genes encoding inhibitors of differentiation (ID) as well as genes that are usually expressed by highly differentiated smooth muscle cells. The induction of these genes was further confirmed at the mRNA level (Northern blot analysis) and the protein level (Western blot analysis) for primary cultures of adult lung fibroblasts. The potential relevance of these observations *in vivo *was established in a separate set of confirmational experiments in rats where it was revealed an overexpression of the ID by myofibroblasts within the fibrotic regions. These novel suggestions have major impact on our understanding of the crucial role of TGF-β1 as a fibroblast differentiation factor in response to fibrogenic stimuli. Nonetheless the present study does not determine the precise role of ID in regulating fibroblast responses to TGF-β1. To do so this study should be coupled with independent methods using ID blocking strategies. Moreover the use of incomplete arrays that detect only the included genes, the variability of cellular composition of tissues studied even from the same organ and the inability of this technology to distinguish changes in cellular composition from transcriptional changes pose major limitations to the global application of these results. Furthermore these observations offer plausible explanations for the lack of similar effects of TGF-β1 in previous studies.

Moreover, bleomycin-induced pulmonary fibrosis rat model was extensively studied by Liu et al. [26] with the use of rat cDNA microarray platforms, in an attempt to highlight genes that may be involved in fibrosis. Interestingly a novel and unexpected finding of this microarray analysis was the identification of FIZZ1 as the most highly and prominently induced gene in bleomycin-treated lungs, evidence consistent with the RT-PCR results. Further analysis of its protein product, demonstrated a unique pattern of localization primarily to alveolar epithelial cells (AECs) derived from bleomycin-injured lungs. To illuminate the exact role of FIZZ1 in inflammation and fibrosis, the effects of co-culturing FIZZ1-expressing AECs on fibroblasts were examined. These analyses demonstrated the significant higher stimulation of normal lung fibroblasts, by high FIZZ1-expressing-AECs, as compared to that observed by control AECs. The major contribution of this new molecule in the differentiation of fibroblasts to myofibroblasts was suggested and verified by its transfection into normal lung fibroblasts which provoked their stimulation independently of TGF-β activation.

These preliminary results suggest that this technology could identify unexpected molecular participants in IPF and might help in the development of novel targets for improved treatment. The method may also allow molecular fingerprinting that could improve the ability to identify subclassifications of pulmonary fibrosis that might be more informative than the current classification based primarily on histologic and radiographic patterns [27]. Nonetheless these studies characterized as "fishing expeditions" are limited by the inability of microarrays to detect the final expression product (protein), identify genes that are not included in the array and ascribe changes in gene expression in specific cellular types. However our view is that there is nothing wrong with a "fishing expedition" if what you are after is "fish", such as new genes involved in a pathway, potential drug targets or expression markers that can be used in predictive or diagnostic fashion. Hence, these observations are not to diminish their value for understanding basic biological processes and even for understanding, predicting and eventually treating human disease (Table 1).

#### 2. Microarrays in asthma (Tables 2 and 3)

**Table 2:** Studies utilizing cDNA microarray technology to study asthma

Investigator	Microarray type	Species/Sample size	Summary/Key findings	Normalization procedure
	Number of genes	Type of tissue		Replications per data point
Zou et al.^32^	cDNA 40.000 elements	10 monkeys Lung tissue samples	Microarray profile of differentially expressed genes in a monkey model of allergic asthma.	Ratios of Cy5/Cy3 multiplied to the balance coefficient of the microarray / 3 replicates
Brutsche et al.^33^	cDNA 600 genes	40 subjects Mononuclear cells	CAGE score for atopy and asthma.	Absolute difference of the expression of CAGE scored genes 1 replicate
Sayama et al.^38^	cDNA 14.000 genes	human umbilical cord mast cells	Transcriptional response of human mast cells stimulated via the Fc (epsilon) RI and identification of mast cells as a source of IL-11.	Array-specific normalization coefficient was calculated by centering in log base 2 space a dataset consisting of all elements with an I/D> 3-fold / 2 replicates
Brutsche et al.^41^	cDNA 600 genes	40 subjects Mononuclear cells	Apoptosis signals in atopy and asthma measured with cDNA arrays	G.I was normalized to the housekeeping G.I / 1 replicate
Syed et al. ^42^	cDNA 12.228 genes	Human CD4^+ ^T cells	CCR7 down-regulation in asthma	Median G.I of each filter normalized any differences in cDNA probe activity between filters/ 1 replicate
Banerjee et al.^43^	cDNA 1.176 genes	18 mice Lung tissue samples	Gene expression profiling in inflammatory airway disease associated with elevated adenosine	G.I was normalized to the housekeeping G.I / 2 replicates

Abbreviations: CAGE: Composite atopy gene expression, CCR7: Chemokine receptor 7, G.I: Gene Intensity, I/D: Intensity/Background ratio, Th: T helper, RI: Immunoglobulin receptor

**Table 3:** Studies utilizing oligonucleotide microarray technology to study asthma

Investigator	Microarray type	Species/Sample size	Summary/Key findings	Normalization procedure
	Number of genes	Type of tissue		Replications per data point
Lee et al.^34^	Oligonucleotide 6.500 genes	Human airway cells	IL-13 induces dramatically different transcriptional programs in three human airway cell types.	Gene expression levels normalized by a scaling factor multiplied to the average of differences of probe pairs (matched-mismatched)/ 1 replicate
Temple et al.^35^	Oligonucleotide 6800 genes	Human eosinophils	Microarray analysis of eosinophils reveals a number of candidate survival and apoptosis genes.	Geometric mean of the scaling (standard experiment) factor served as normalization factor/ 2 replicates
Hakonarson et al.^36^	Oligonucleotide 5.000 genes	Rabbit and human ASM	Association between IL-1beta/TNF-alpha-induced glucocorticoid-sensitive changes in multiple gene expression and altered responsiveness in ASM.	Gene expression levels normalized by a scaling factor multiplied to the average of differences of probe pairs (matched-mismatched) / 2 replicates
Laprise et al.^37^	Oligonucleotide 12.000 probe sets	8 subjects Lung tissue samples	Functional classes of bronchial mucosa genes that are differentially expressed in asthma.	Mean hybridization intensities of all probe sets on each array were scaled to a fixed level/ 2 replicates
Nakajima et al.^39^	Oligonucleotide 12.000 genes	Human MCs and eosinophils	Gene expression screening of human mast cells and eosinophils using high-density oligonucleotide probe arrays: abundant expression of MBP in MCs	Mean hybridization intensities of all probe sets on each array were scaled to an arbitrary, fixed level / 1 replicate

Abbreviations: ASM: Airway Smooth Muscle cells, MBP: Major Basic Protein, MCs: Mast cells, TNF: Tumor Necrosis Factor

Asthma is one of the most serious allergic diseases associated with both genetic and environmental factors such as allergens, respiratory tract infections, and atmospheric pollutants. Most asthma is associated with atopy, a predisposition to generate immunoglobulin (Ig)-E against environmental allergens [28]. However, only a proportion of atopic individuals develop lower airways symptoms consistent with an asthmatic phenotype. It is therefore tempting to speculate that the development of asthma requires combined inheritance of genes which alter the immune cell response to the environment, and at the same time, render the airways structural and neural regulation susceptible to injury caused by inflammation. Several asthma/atopy associated genes have been identified from linkage and association studies within families and revealed that there are multiple chromosomal regions, containing potential candidate genes, associated with various asthma phenotypes [29-31].

Microarray technology offers a new opportunity to gain insight into global gene expression profiles in asthma, leading to the identification of asthma associated genes. Several experimental models have been used for this purpose although no animal disease model is identical to human disease. Zou et al.[32] were the first attempted to produce an allergen-induced gene expression profile in the lung of a non-human primate using genomics tools such as microarrays and real time-(RT)-PCR in an independent way. Microarray data generated from this study and validated by RT-PCR using same lung samples, revealed a differential gene expression pattern between control and challenged animals. Furthermore investigators established that genes identified by microarray technology represented genes truly regulated by inhalation antigen challenge. This was done by determining that the regulated expression levels identified by microarray assay from a single animal were confirmed by RT-PCR studies using multiple similarly treated animals. Potential limitations of this study include the time-limited gene expression profile tested which may not reflect the chronic aspect of asthma and the absence of evidence that the antigens used would produce the same allergic reaction in humans.

Brutsche et al. [33] designed an array based composite atopy gene expression (CAGE) score to evaluate the diagnosis of atopy and asthma and assess disease activity in order to guide therapeutic decisions. The CAGE score was determined by using 10 genes dysregulated atopic individuals according to a specific algorithm. The application of this score in a group of asthmatic patients revealed that this approach had a better sensitivity and specificity than total IgE in differentiating atopic from non-atopic subjects. Correlation between CAGE score and total IgE was found, and there was a trend for correlation with asthma severity. It is noteworthy that the CAGE score was able to quantify phenotype-specific alteration in gene expression of atopic individuals. Perspectively the CAGE score can be further improved through a better reproducibility of microarray systems compared with the filter arrays and the possibility of a better selection of genes. Therefore it may be used as a prognostic and diagnostic tool or to monitor the effects and side-effects of asthmatic therapy in the not distant future.

Several morphologic changes in the airways of patients with asthma have been attributed to the Th-2 produced cytokines such as IL-13 and IL-5. However the molecular mechanisms underlying the contributions of these cytokines to asthma remain largely unknown.

Towards this direction Lee et al. [34] applied oligonucleotide microarray technology in primary cultures of three human airway cell types (epithelial, smooth muscle cells and lung fibroblasts) to elucidate the effects of IL-13 in these cell types. Interestingly, the results of this study demonstrated that despite initiation of an identical signaling pathway (STAT6), IL-13 induced highly distinct transcriptional programs in each of the three cell types suggesting a coordinate and distinct contribution to asthma pathogenesis by each of the cell types examined. Although the quality of the genechip analysis was estimated and validated by RT-PCR methods applied in a small number of selective genes, however there are important limitations in this study including the possible differences between transcriptional responses and gene expression profile of a cell type *in vivo *and *in vitro*.

One of the greatest disadvantages of microarrays and at the same time challenges for most of the investigators is the objective difficulty dealing with the results of the experiments resulting from the large quantities of information. Currently, the hurdle faced is the routine interpretation of this information to identify among thousands of dysregulated genes, those who are informative, causal and specific to the phenotypic change of interest. Thus, Temple et al. [35] compared the results derived from the application of oligonucleotide microarray technology in eosinophils isolated from human peripheral blood before and after treatment with IL-5 and in an alternative cellular model, TF1.8 cells, whose survival was known to be dependent on IL-5. Comparison of these two models facilitated the identification of the genes that rule the apoptosis and survivability of eosinophils and demonstrated a small group of genes whose regulation was similarly coordinated in both systems. Authors combined different cellular models focused on the same experimental paradigm and looked for common changes. This approach helped the scientists to focus attention on a subset of genes most likely to be causal and relevant to the phenotypic change of interest and filter out non-specific gene expression change. Combination of this method with proteomics approaches and tissue distribution analysis can add another filter for genes of interest and generate data of sufficient scientific rigidity.

Microarrays apart from their remarkable effectiveness in identifying novel gene expression patterns can also be used to clarify physiological mechanisms underlying the actions of numerous drugs, such as those applied in the management of patients with asthma. Several studies have utilized microarray technology to assess the gene expression profile of cells and tissues before and after treatment with commonly applied drugs such as corticosteroids. Two of them are reviewed here.

Recently, Hakonarson et al. [36] in addition with the role of two pleiotropic cytokines, IL-1β and tumor necrosis factor (TNF)-α, in the pathophysiology of asthma, have reported the effectiveness of dexamethasone in the treatment of asthma with the use of oligonucleotide microarrays. The accumulation of the two cytokines in a medium where human airway smooth muscle-(ASM) – cells were cultured, elicited an overexpression of several proinflammatory genes known to regulate smooth muscle contractility and relaxation. The latter finding noted in four separate microarray experiments was consistent with the increased responsiveness of rabbit cytokine-treated tissues in acetylcholine. The administration of dexamethasone provoked a repression to the majority of the microarray-study derived genes as well as to the contractility of the cytokine-treated ASM. Collectively these findings suggest a crucial role of ASM in expressing a host of glucocorticoid-sensitive proinflammatory gene patterns that affect the structure and the function of the airways. However these observations were based on studies using rabbit and human ASM cells. Hence the issue of potential species differences warrants consideration. Further studies using samples derived from homogeneous species and validation techniques are necessary to streamline these observations.

Microarray analysis performed by Laprise et al. [37] indicated a differential gene expression pattern in bronchial tissues from healthy and asthmatic individuals, a profile that included not only genes previously implicated in the pathogenesis of asthma but also new potential candidates. The remarkable ascertainment of this study, conducted with bronchial tissues which are known as a primary site for airway inflammation and remodeling, was that the expression of one third of the genes was partially or completely corrected by inhaled corticosteroid treatment. The latter evidence further illuminates the true impact of first line therapy offered to asthmatic patients. However application of this technology may be limited by the disease's spatial and temporal heterogeneity due to differences in cellular composition between asthmatic and control tissue. Ultimately the results obtained using microarrays need to be verified firstly with confirmational studies (RT-PCR and in situ hybridization) and secondly with separate experiments.

Mast cells represent key cells in the initiation and progression of asthma, releasing several mediators of inflammation, such as certain cytokines and chemokines. The past few years several studies have been focused on the identification of new mast cell products through the gene expression analysis. In one of them published by Sayama et al. [38] application of cDNA microarrays in only two populations of stimulated human mast cells exhibited among other genes a significant upregulation of the gene encoding IL-11. The latter finding was further confirmed by a separate set of experiments where an increased secretion of IL-11 by activated human mast cells was noted. However further microarray analyses coupled with functional approaches and independent studies examining the potential role of IL-11 in the pathogenetic mechanisms of asthma as well as in the alterations of mast cell proliferation and survival, are required.

Furthermore, Nakajima et al. [39] in their attempt to evaluate the significance of protein products present in mast cells applied oligonucleotide microarray technology in human mast cells derived from different sources and in eosinophils. The most impressive finding of this study was the abundant expression of major basic protein (MBP) among the transcripts for expected mast cells specific proteins such as tryptase. Authors also confirmed in independent studies using RT-PCR and flow cytometry that MBP was expressed at both the transcript and protein levels in various types of mast cells. While this result is really intriguing and opposes to the already known data which indicates the unique presence of MBP to eosinophils [40], it is incomplete and unable to determine the biologic significance of MBP present in mast cells.

Microarrays using nylon membrane radioactive cDNAs have already been applied in the research field of asthma and much good work has been done with this technology.

Brutsche et al. [41] applied nylon membrane cDNA microarray technology in blood samples derived from atopic asthmatic and nonasthmatic patients, and healthy control subjects to investigate the systemic activation of apoptosis pathways of inflammatory cells in lung tissue. They identified significantly altered expression of several apoptosis-related genes in atopy and asthma compared with the healthy subjects suggesting that these alterations could be due to genetic or environmental factors. Several verification experiments have been used to further validate a considerable amount of differentially expressed genes on mRNA level (RT-PCR), as well as protein (ELISA) and receptor level (double stained fluorescence methods). The profile of altered gene expression did not show a definite pattern that was suggestive of survival or apoptosis. Potential criticisms of this approach include the large amounts of data variability derived from the heterogeneity of the studied samples and the lack of proteomics analyses. Thus this study is unable to give any statement on the activity of the apoptotic pathways. To do so the data should be combined with the proteomics analysis of proteins involved in apoptosis. The latter will contribute to the characterization of protein patterns and will allow for the assessment of overall changes in the protein content associated with apoptosis.

One of the first gene-profiling studies highlighting the potential role of chemokines and their receptors in the pathogenesis of asthma was conducted by Syed et al. [42] They used nylon membrane radioactive arrays compiled from mixed biological samples to determine the gene expression pattern of T-cells from patients with atopic and non-atopic asthma and found altered gene expression profile for CCR7 (chemokine receptor 7) between patients and controls, findings that were confirmed by RNA dot plot analysis. Data derived from this analysis is indicative of a possible role of this molecule in the progression of asthma. However the small number of patients recruited in this study and the lack of functional genomic analysis allow us to make only speculations on the exact role of this factor in the disease process.

In another study Banerjee et al. [43] in their attempt to identify and characterize biological roles for adenosine-regulated genes applied radioactive cDNA microarray technology in lung specimens derived from normal and adenosine deaminase (ADA)-deficient mice. The results of this study profile the differential expression of a vast number of genes, that may be regulated by adenosine and hence play a pivotal role in modulating the underlying lung pathology. The reliability of the results derived from the microarray approach was also confirmed with gene specific RT-PCR analysis. Moreover authors used a separate set of experiments and demonstrated both with microarray analysis and protein localization that therapy with ADA in the deficient group of mice regulated expression of several genes modulating pulmonary inflammation and cell adhesion. The consistency of findings derived from the two experiments provides to microarray analysis a high degree of confidence. However critical limitations of this study originate from the disability of gene expression analysis to distinguish changes in transcriptional regulation from changes in cellular composition. Hence a more in-depth analysis is required to quantify the gene expression and establish a direct regulation of these genes by adenosine signaling.

Accumulated evidence from these analyses revealed that microarray analysis can be a powerful tool for identifying mediators of allergic asthmatic disease through a genomic-based strategy using non-human primates and provide us a novel large scale of differentially expressed genes. Additionally, authors compared different cellular models sharing similar experimental paradigm to focus on the most likely informative genes and filter out the bystanders. The application of this approach further streamlined the pivotal role of microarrays in determining transcriptional responses of genes to several inflammatory cytokines and in identifying gene expression patterns and important mediators associated with the initiation and the progression of asthma. Moreover, microarrays coupled with separate set of experiments have provided the investigators with useful knowledge concerning the efficacy of the already applied drugs in the treatment of asthma and helped them to understand their anti-inflammatory role in terms of physiology and molecular biology. Nevertheless, most of the studies exhibited essential weaknesses generated by the heterogeneity of samples studied and compared and by the disability of microarrays to quantify gene expression and yield information about transcriptional responses and post-translational protein modifications. Therefore more in depth analysis of the microarray results is needed in combination with novel approaches that will help us focus on the specific genes and elucidate their role in the cellular function and the pathogenesis of asthma (Tables 2,3).

#### 3. Microarrays in Chronic Obstructive Pulmonary Disease (Table 4)

**Table 4:** Studies utilizing microarray technology to study COPD

Investigator	Microarray type	Species/Sample size	Summary/Key findings	Normalization procedure
	Number of genes	Type of tissue		Replications per data point
Koike et al.^45^	cDNA 450 genes	Rats AM	cDNA microarray analysis of gene expression in rat alveolar macrophages in response to organic extract of diesel exhausts particles.	G.I was normalized to the housekeeping G.I 2 replicates
Yamanaka et al.^46^	cDNA 18.432 genes	Human AEC	Gene expression profiles of human small airway epithelial cells treated with low doses of 14- and 16-membered macrolides.	G.I was normalized to the housekeeping G.I 3 replicates
Fuke et al.^47^	cDNA 77 genes	30 patients Lung tissue specimens	Chemokines in bronchiolar epithelium in the development of chronic obstructive pulmonary disease.	Signal normalized to a given gene transcript 3 replicates
Vuillemenot et al.^48^	Oligonucleotide 12.000 genes	10 mice Lung tissue specimens	Lymphoid tissue and emphysema in the lungs of transgenic mice inducibly expressing tumor necrosis factor-alpha.	Signal normalized to internal control 2 replicates
Hackett et al.^50^	cDNA 4.600 genes	22 individuals AEC	Variability of antioxidant-related gene expression in the airway epithelium of cigarette smokers.	Mean hybridization intensities of all probe sets on each array were scaled to an arbitrary, fixed level / 2 replicates
Morris et al.^52^	Oligonucleotide 6.500 genes	Mice Lung tissue samples	Loss of integrin alpha (v) beta6-mediated TGF-beta activation causes MMP-12-dependent emphysema.	Mean hybridization intensities of all probe sets on each array were scaled to an arbitrary, fixed level / 2 replicates
Golpon et al.^53^	Oligonucleotide 6.500 genes	Human/mice Lung tissue samples	HOX genes in human lung: altered expression in primary pulmonary hypertension and emphysema.	Gene expression levels normalized by a scaling factor multiplied to the average of differences of probe pairs/ 3 replicates

Abbreviations: AEC: Airway Epithelial Cells, AM: Alveolar Macrophages, COPD: Chronic Obstructive Pulmonary Disease, G.I: Gene Intensity, TGF-b: Transforming Growth Factor-beta, MMP: Metalloproteinase

Chronic obstructive pulmonary disease (COPD) is a chronic disease characterized by progressive airflow obstruction, chronic cough and dyspnea in advanced stages, caused by smoking, environmental, and hereditary factors. It is associated with two clinical entities, chronic bronchitis and emphysema. In nowadays, the invention and application of microarray technology offers scientists the opportunity to gain a better understanding on the pathophysiology of COPD through the identification of novel gene expression patterns, leading to illumination of genes candidates for modern therapeutical approaches [44].

It is already known that chronic bronchitis can be induced by several types of environmental pollutants such as diesel exhaust particles (DEP). Though recently a microarray study has been published by Koike et al. [45] addressing the effect of such pollutants on the gene expression profiles of alveolar macrophages, however a complete analysis including the transcriptome and proteome, is needed to elucidate the toxic effect of air pollutants on pulmonary cells.

Microarray approach is already being applied in respiratory clinical pharmacology with the identification of genes {Yamanaka et al. [46]} that can serve as potential molecular targets of common drugs applied in the management of patients with chronic bronchitis. However, studies being published in the field of respiratory pharmacogenomics lack of scientific rigidity primarily due to incomplete available arrays that will help scientists to determine much larger numbers of pharmacologically relevant genotypes. Far more progress should be made towards this direction.

One of the major limitations in our attempt to elucidate the exact role of specific cell types in the pathogenesis of COPD is the compact anatomy of the lung which makes unraveling specific cell type gene expression changes difficult, requiring immunoelectron microscopy or laser capture microdissection. The first study to perform quantitative cell type-specific gene expression analysis using the pioneering technology of laser capture microdissection in human tissue samples coupled with RT-PCR and cDNA approach was recently published by Fuke et al. [47] Authors performed individual analyses and revealed a specific cell type upregulation of three inflammatory chemokines reportedly relevant to the pathogenesis of COPD emphasizing the pivotal role of these cells and their products in driving the inflammation. Although data was not fully confirmed by microarray analysis, however discrepancies between methods illustrate more the potential danger of depending solely on array approach rather than limit the scientific consistency of these results. Further research investigating the functional consequences of these changes is required.

Several inflammatory cytokines have been implicated in the pathogenesis of emphysema, including TNFa, a molecule with versatile pathogenetic mechanisms. As a means to investigate some of them that culminate to lung-pathology, Vuillemenot et al. [48] applied oligonucleotide microarray technology coupled with independent studies (histologic and immunohistochemical analyses) in an experimental model they developed. Results derived both by microarray approach and independent studies revealed a direct correlation between TNFa and emphysema. However functional approaches should be applied in combination with gene expression analysis to shed further light in the mechanisms by which TNF promotes airspace enlargement.

Despite the clear link of smoking to the risk for chronic bronchitis, only 15–20% of cigarette smokers develop COPD, [49] suggesting that there must be risk factors other than smoking that contribute to the susceptibility to this disease. To address this issue Hackett et al. [50] implemented microarray technology in human airway epithelial cells of smokers and non-smokers and demonstrated differential anti-oxidant related gene expression between the two groups of volunteers. One of the most intriguing aspects of this study is that individual assessment by hierarchical clustering of anti-oxidant related gene expression in response to smoking displayed a remarkable variability suggesting variability in the responses of different individuals to the chronic oxidant stress of smoking. However the extent of this variability may be explained by the nature of the microarray assay and the large amounts of data variation derived from these studies. Thus it is of high risk to speculate that these genes may serve as useful genetic markers in future epidemiologic studies determining susceptibility to smoking induced COPD. Further studies applying high-tech computational clustering tools coupled with independent validation tests are required.

One of the most exciting aspects of microarrays is their use as tools for actively introducing serendipity to one's research [51]. In experiments designed to identify global transcriptional programs responsible for regulating lung inflammation and pulmonary fibrosis, as described previously, [23] microarray experiments were performed by Morris et al. [52] on lung tissue from wild-type mice and mice lacking a member of the integrin family (avβ6) known to be involved in activation of latent TGF-β. In addition to identifying distinct cluster of genes involved in these processes, these studies combined with RT-PCR validation tests and independent experiments led to the identification of novel pathways by which TGF-β can regulate emphysema through the upregulation of Mmp-12, the most highly induced gene in the lungs of β6 knockout mice. The role of Mmp-12 in the pathogenesis of emphysema was verified in an independent cohort where β6 knockout mice deficient in the expression of Mmp-12 displayed no alveolar enlargement. Although these results do not eliminate the possibility that other proteases may interact with Mmp-12 in the development of emphysema, however suggest that abnormalities in any of the steps in this pathway of TGF-β activation may contribute to genetic or acquired susceptibility to emphysema in humans.

Presently, very few studies dealing with the role of HOX genes in the adult respiratory system are available in the literature. Golpon et al. [53] investigated the expression pattern of HOX genes, in fetal and diseased lung specimens (emphysema, primary pulmonary hypertension), by applying two microarray survey techniques and their analysis reflects one of the most detailed and informative studies in this field. They compared the HOX gene expression pattern in human and mouse lungs and found that HOX genes are selectively expressed in the human lung. This study also yielded an altered HOX-gene expression pattern among fetal, adult and lung specimens with emphysema and pulmonary hypertension, by identifying different types of HOX genes overexpressed in each of these conditions indicating differential HOX gene expression as a potential factor that contributes to the development of certain pulmonary diseases. Though the overall size of tissue samples studied was small data from this study comprises evidence with high degree of confidence, validated and confirmed both in an independent cohort (degenerate RT-PCR) and by alternative methods (quantitative RT-PCR and in situ hybridization). Possible limitations include small number of tissues studied, incomplete microarray survey techniques and minor discrepancies between the findings generated from validation studies.

Collectively these results suggest that microarray analysis with its ability to highlight gene expression profiles on a large scale and coupled with progressive technologies and independently validated data has led researchers to shed further light into transcriptional programs regulating emphysema and to the identification of common mediators and molecular pathways involved in the pathogenesis of both COPD and pulmonary fibrosis, indicating novel targets for therapeutic interventions and useful genetic markers assessing susceptibility to COPD. Although limitations such as inconsistency between findings derived by microarray approach and independent studies, lack of functional changes assessment and significant data variability can be detectable in these studies, however evidence derived from these analyses is valuable and heavily informative (Table 4).

#### 4. Microarrays in acute lung injury and pulmonary edema (Table 5)

**Table 5:** Studies utilizing microarray technology to study ALI/PE

Investigator	Microarray type	Species/Sample size	Summary/Key findings	Normalization procedure
	Number of genes	Type of tissue		Replications per data point
McDowell et al.^56^	cDNA 8.374 genes	6 mice Lung tissue samples	Differential gene expression in the initiation and progression of nickel-induced ALI.	Ratios of Cy5/Cy3 multiplied to the balance coefficient of each microarray / 5 replicates
Olman et al.^58^	cDNA 588 genes	36 patients Pulmonary edema Lung fibroblasts	Microarray analysis indicates that pulmonary edema fluid from patients with ALI mediates inflammation, mitogen gene expression, and fibroblast proliferation through bioactive IL-1.	G.I normalized to the housekeeping G.I 2 replicates
Kupfner et al.^61^	Oligonucleotide	Mice Lung neutrophils	Role of NF-κB in endotoxemia-induced alterations of lung neutrophil apoptosis.	Gene expression levels normalized by a scaling factor multiplied to the average of differences of probe pairs / 3 replicates
Cher et al.^62^	Oligonucleotide 8.800 genes	21 rats Whole lungs	Pulmonary inflammation and edema induced by Phospholipase A_2._	Each gene was divided by the median of its values in all samples / 3 replicates
Sabbadini et al.^63^	Oligonucleotide 12.600 genes	14 rabbits Lung tissue samples	Gene expression analysis in interstitial lung edema induced by saline infusion.	Gene expression levels normalized by a scaling factor multiplied to the average of differences of probe pairs / 2 replicates
Perkowski et al.^64^	cDNA 8.374 genes	20 mice Lung tissue samples	Gene expression profiling of the early pulmonary response to hyperoxia in mice.	Difference between observed log-ratio and corresponding fitted ratio/ 5 replicates
Ward et al.^65^	cDNA 7.398 genes	6 rats Lung and other organ samples	Molecular signatures of sepsis: multiorgan gene expression profiles of systemic inflammation.	Gene expression levels normalized by a scaling factor multiplied to the average of differences of probe pairs / 4 replicates

Abbreviations: ALI: Acute Lung Injury, G.I: Gene Intensity, PE: Pulmonary Edema, NF-κB: Nucleus Factor-κB

Acute lung injury (ALI), a severe respiratory syndrome, develops in response to numerous insults. This syndrome that responds poorly in therapeutic interventions and has a poor prognosis has been associated with a myriad of mediators including cytokines, reactive oxygen and nitrogen species, growth factors and proteolytic enzymes [54,55]. Despite extensive research since the initial description of ALI over 30 yr ago, questions remain about the basic pathophysiologic mechanisms that are critical to the diminished survival and their relationship to therapeutic strategies. McDowell et al. [56] in their attempt to determine the interactions between the great amount of factors that have been associated with the development of ALI, analyzed 8,374 murine cDNAs for temporal changes and functional relationships throughout the initiation and progression of ALI in mice exposed to particulate NiSO_4_. Novel interactions between factors (antioxidant genes) previously associated with ALI and factors (surfactant proteins) previously not associated with ALI emerged from the application of functional genomics during nickel-induced ALI. Data derived from this experiment and partially confirmed by Northern blot analysis and nuclease protection assays is valuable and consistent with the ongoing attempts to treat ALI with exogenous surfactant-associated proteins [57] in combination with antioxidant therapy and may determine new therapeutical interventions. This study reveals the great importance of functional genomics not simply to provide a catalogue of all the genes and information about their functions, but to help scientists to understand the possible interplay of components contributing to lung injury.

Although the fibroproliferative response to lung injury occurs in high frequency in patients with ALI, the mechanisms of this response are largely unknown. One of the most meaningful and informative studies addressing this important issue was recently published by Olman et al [58]. Authors applied radioactively nylon membrane arrays in human lung fibroblasts exposed either to ALI or hydrostatic pulmonary edema fluids and revealed a potential mitogenic activity of IL-1β and its importance as a modulator of fibroblast proliferation. Data derived from this study of a considerable number of patients and replicated both in an independent cohort (use of IL-1 antagonist receptor) and by alternative laboratory methods (RT-PCR, Northern blot analysis) is of fundamental value and may provide scientists with several independent lines of evidence that IL-1 amplifies the inflammatory and fibroproliferative process through regulation of fibroblast mitogenesis and gene expression. A major limitation mentioned in this study was the pooling of pulmonary edema samples due to their limited volume that did not allow authors to perform clinical-molecular correlations in an individual way.

ALI is frequently associated with endotoxemia and is characterized by the accumulation in the lungs of large populations of neutrophils activated to produce proinflammatory mediators. Many studies had demonstrated a critical role of the endotoxemia-induced activation of NF-κB in neutrophils in the development of ALI [59,60]. One of the most important studies focused on the role of NF-κB activation in lung neutrophils apoptosis after endotoxemia was conducted by Kupfner et al. [61]. Though gene expression analysis revealed a significant role of NF-κB as a modulator of neutrophil apoptosis and data was confirmed by proteomics analysis there were major discrepancies between these findings and the results derived from individual experiments utilizing an inhibitor of nuclear translocation of NF-κB. The latter demonstrated no significant alterations in the percentage of the endotoxemia -induced apoptotic lung neutrophils in mice treated with the inhibitor evidence that reflects anti-apoptotic mechanisms not solely dependent on NF-κB. Although these findings can be informative indicating novel anti-apoptotic pathways for modern therapeutic approaches they should be re-evaluated in the context of new microarrays analyses of considerable sample size coupled with confident validation steps and independent experiments.

To gain a more comprehensive understanding on the complex interplay between several inflammatory cytokines involved in the pathogenesis of pulmonary edema, several group of investigators {Cher et al. [62], Sabbadini et al.[63]} applied oligonucleotide microarray techniques in different experimental models of lung edema. Interestingly, these analyses revealed differential expression patterns for many inflammatory genes implicating some of them in the pathogenesis of pulmonary edema. Though these studies applied several confirmational tests (RT-PCR, Northern blot, Western blot) to validate results derived form microarray experiments, there are substantial weaknesses and concerns such as discrepancies between findings determined by these techniques that pose major limitations and limit their scientific rigidity.

The study of Perkowski et al. [64] has been on of the most extensive and informative studies of the effect of 100% oxygen on the mouse lung. The authors used the cDNA microarray approach to evaluate the molecular profiling occurring during the early response to hyperoxia in mice. Among the vast amount of data derived from two different array sets they distinguished a cluster of genes of great interest (antioxidant enzymes, cell cycle progression regulators, endothelial cell and ECM genes) whose expression was substantially altered in response to hyperoxic stimuli. Authors were encouraged to observe that, of those genes where array data was compared with RT-PCR, changes occurred in same direction and were of similar magnitude. Further validation of the data with Northern-blot analysis for some of these genes reassuringly confirmed these alterations whereas for only one gene investigators performed functional activity assessment demonstrating similar notable findings. Though the results of this microarray study were double validated by standard molecular biology techniques there are still a number of caveats that should be kept in mind, including the post-transcriptional modifications that cannot be readily detected by gene expression changes and the large amounts of data variability that limits the careful analysis of every gene and highlights the necessity for further confirmatory tests and studies utilizing human lung samples.

One of the first gene-profiling studies to address an important disease process, such as sepsis at a multisystem level, was that of Ward et al. [65] Using DNA microarray platforms, authors examined the sepsis-induced gene expression patterns at a multiorgan level, in mice. One of the most intriguing aspects of this study is the identification of genes (many of them not previously associated with sepsis response) that have a distinctive organ-specific expression profile as well as of genes with a relatively universal response to sepsis indicating interesting associations between organs. Validation of the data was performed by Northern blot analysis in only four selected genes and similar quantitative concordance between the two analyses was achieved. Although the microarray analysis provides an informative insight in the pathogenetic mechanisms of a complex disease process the lack of sufficient confirmatory tests applied in all studied genes and the absence of functional genomics that will help us understand the exact role of the newly characterized genes in the septic response pose major limitations in the study and illuminates the need for further characterization of the sepsis-induced gene expression profiles.

In summary, these studies exhibit the crucial role of a novel molecular technology in discovering, through global analysis of gene expression, genes previously identified only by their DNA sequence. Although the array analysis provides in some studies a comprehensive overview of gene expression in the lung during ALI [56,58,61,64], and sepsis [65] and after hyperoxia [64], however there are numerous concerns arising from the large amounts of data variability, the lack of proteomics approaches in most of them and the controversial findings of microarray analysis and confirmational techniques. Unfortunately only three studies [58,61,64] used independent methodological criteria to validate a relatively small portion of their results, evidence that highlight the necessity for further more widespread evaluation of these findings. With the use of these approaches, more precise diagnosis and risk assessment of ALI based on expression profiles can be achievable in the next ten years, leading to more accurate determination of prognosis and new therapeutical interventions (Table 5).

#### 5. Microarrays in sarcoidosis

Sarcoidosis is a chronic systemic disorder characterized by the presence of non-caseating granulomas and accumulation of T-lymphocytes and macrophages in multiple organs [66]. The mechanisms leading to the persistent accumulation of inflammatory cells are not fully understood. Apoptosis, a dynamic process involved in the control of the "tissue load" of immune effecter cells at inflamed sites, limits inflammatory tissue injury and promotes resolution of inflammation [67]. Whether or not reduced apoptosis is involved in the pathogenesis of sarcoidosis is unclear. Rutherford et al. [68] in their attempt to shed further light on apoptosis signals in the peripheral blood of sarcoidosis patients with self limited and progressive disease in comparison with healthy controls used high-density probe arrays containing 12.626 genes. Though this study demonstrated significant differences in the expression of apoptosis-related genes in peripheral blood of patients with acute onset sarcoidosis compared to controls, ultimately did not manage to show a definite profile that was suggestive of survival or apoptosis. Although authors applied functional genomics a potential criticism of their approach is that they cannot give any statement on the activity of the apoptotic pathways. To do so the data should be combined with the proteomics analysis [69] of proteins involved in apoptosis. The latter will contribute to the characterization of protein patterns and will allow for the assessment of overall changes in the protein content associated with apoptosis.

Collectively these findings not only reveal the importance of the microarray platforms in identifying gene expression patterns that give the scientists the opportunity to elucidate the pathophysiological processes of complex diseases, such as sarcoidosis but also illuminate some of their origin disadvantages.

### Future directions, challenges and limitations of microarray technology

The last five years has seen the emergence of a novel technology applied in almost every aspect of respiratory research, a technology that has also great future perspectives and may provide scientists with numerous avenues of investigation that have clinical implications. Since nowadays, microarray technology has been successfully used for the identification of potential target genes for therapeutic intervention in IPF,[22] mechanistic studies in animal models of asthma [32,45] and IPF,[23,24] and helped the investigators to shed further light into the transcriptional programs involved in cytokine signaling [34,36,42] and apoptosis [35,41,61]. Furthermore, this technology increased our hopes in the field of diagnosis and clinical assessment of complex diseases such as asthma [33] and revealed modern approaches in therapeutic interventions in asthma [37,43] as well as COPD [46]. Finally, it provided scientists with useful information in their attempt to gain better understanding in molecular mechanisms regulating several pathological processes such as IPF, [22-26] asthma, [33,35-39,42] COPD, [45-48,50,52] lung fibrosis in acute lung injury [56,61,64,65] and pulmonary edema [58,63,64]. However, the feeling of excitement arising from the relative ease of producing the results of microarray experiments comes to contrast with the confusion arising from the objective difficulty of dealing with the results.

Managing and mining the huge amount of data generated by microarray experiments still remains a major challenge and limitation for most investigators. Whether gene expression changes are considered primary vs. reactive for a given disease is a complicated issue, and one can only begin to judge that if the methods and approaches used to generate the data are of sufficient scientific rigidity. The diversity and scope of the data require the creation of multidisciplinary teams consisting of physicians, biologists and bioinformaticians (mathematicians, computational biologists and database managers) [10]. Thus, we can conclude, that these diverse experimental schemes pose diverse computational requirements, such as advanced data mining, clustering and analysis tools, including interpreting patterns of gene expression with self-organizing maps.

**Figure 4:**
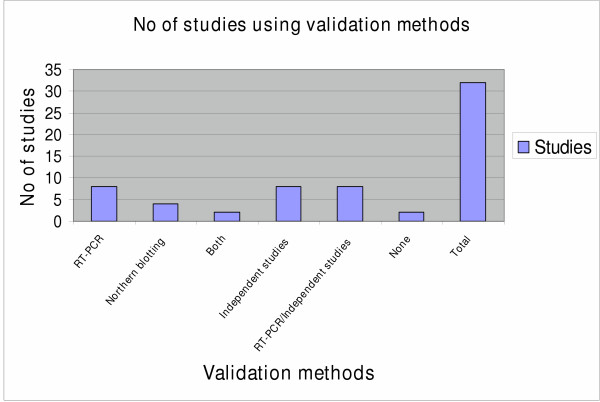
Diagram showing the number of studies cited in this review article that validated the data derived from the microarray analysis either by confirmational studies (RT-PCR, Northern blot analysis, or both) or independent experiments (protein analysis, in situ hybridization, transgenic mice etc) in comparison with the total number of studies reviewed in this article. The majority of the studies cited in this review manuscript have used at least one confirmational test to replicate the microarray findings.

Because of the statistical issues raised by microarray technology, it is necessary for any meaningful interpretation that the data is replicated using independent methodological criteria, preferably with separate samples rather than the tissue or RNA used to derive the original targets. A rapid high through-put method for confirmation of microarray data is quantitative RT-PCR. Alternatively, Northern blots or ribonuclease protection assays provide the benefit of direct quantification. So far some studies have started to adapt these approaches (Figure 4) but there are still limitations. Because a microarray analysis may reveal putative changes in the expression of tens or hundreds of genes, it is practically impossible to validate all of the data. However, it is incumbent upon investigators to evaluate a reasonable number of biased in their selection genes. Thus genomics and gene expression experiments are sometimes derided as "fishing expeditions". Hence it is necessary that these conventional techniques should be coupled with advanced data mining tools to help the scientists to face the greatest challenge, namely the extraction of biological meaning from microarray data and the prioritization of candidate genes for follow up [11]. Another challenge is to gain a holistic view of the human genome and biology, by applying genomic microarray in combination with the proteomic microarray to overpass the origin disadvantage of microarray technology that gives the users the view of inducible genes only. Sequence and gene expression analysis alone is insufficient to fully inform the investigator on the cell state and function. To the best of our knowledge only few of the studies utilizing microarray platforms in respiratory research has taken advantage of this approach and in a limited number of genes [69]. This combination would be crucial to better understand the functional aspects of disease and to bridge the long way between genotype and phenotype due to environment-gene interactions [12,70]. Additionally, it will be critical to develop improved methods for unbiased amplification of small RNA samples so that meaningful data can be obtained by applying microarrays on small tissue samples and pure cell populations, such as are samples obtained by microdissection of tissue sections [47]. This approach will solve the problem created by dramatic differences in cellular composition of affected and unaffected tissue and by spatial and temporal heterogeneity of disease that limits the optimal application of microarrays to the study of diseases [27]. Finally it is noteworthy to be mentioned that microarrays like other new diagnostic and research tools are highly cost-intensive. Considering the high costs of microarray based experiments it is important to say that this disadvantage will inevitably limit the speed with which they are introduced into clinical practice and restrict their application in university hospitals and other medical institutes [71]. Therefore it is crucial that academic centers and other specialized units should understand that joint ventures with biotechnological and pharmaceutical companies are critical to overlap all the financial obstacles so that microarray technology will be likely to reach most large hospitals with huge potential gain in clinically relevant information for individual patients and their diseases.

## Conclusion

Currently, the use of microarray technology in respiratory research is limited by the tissue sample, incomplete available arrays and the analysis of data generated from this technology. Clinical use of microarrays technology is still in its infancy and remains exploratory. For these array-based methods to become truly revolutionary, they must become an integral part of the daily activities of the typical molecular biology laboratory and biomedical institute. There is plenty of room for technical improvements, further development, and more widespread acceptance and accessibility. Optimistically thinking we expect that over the next few years the pattern of development and use of microarrays will be on a similar trajectory to that seen for computers and other high-tech electronic devices, which started out as exotic and expensive tools in the hands of the few developers and then moved quickly to become easier to use, more available and less expensive. Alternatively authors believe that so far microarrays have not added much to our understanding and the possibility to live up to the great 'hype' that was generated belongs to the distant future.

Our view is that the application of these approaches will improve dramatically the effectiveness and reliability of microarray technology in studies of diseases of complex organs like the lung, and will have a major impact on our understanding of molecular pathogenesis for the foreseeable future. Whether our hopes will be fulfilled or disproved remains to be seen.

## List of abbreviations

ADA: Adenosine Deaminase

ALI: Acute Lung Injury

ASM: Airway Smooth Muscle

a-SMA: a-Smooth Muscle Actin

BAL: Bronchoalveolar lavage

CAGE: Composite Atopy Gene Expression

COPD: Chronic Obstructive Pulmonary Disease

CCR7: Chemokine Receptor 7

DEP: Diesel Exhaust Particles

ECM: Extracellular Matrix

eQTL: expression quantitative trait locus

G.I: Gene Intensity

Ig: Immunoglobulin

ID: Inhibitor of Differentiation

I/D: Intensity/Background ratio

IPF: Idiopathic Pulmonary Fibrosis

MBP: Major Basic Protein

MCs: Mast Cells

MCP: Monocyte Chemoattractant Protein

MMP: Matrix Metalloproteinase

NF-κB: Nuclear Factor-Kb

nsPLA_2_: snake venom phospholipase A_2_

RT-PCR: real time-polymerase chain reaction

TGF-b: Transforming Growth Factor-b

TNF: Tumor Necrosis Factor

UIP: Usual Interstitial Pneumonia

VEGF: Vascular Endothelial Growth Factor

## Competing interests

The authors declare that they have no competing interests.

## Authors' contributions

AT, GP and DB were involved with the study conception. AT performed the data acquisition and interpretation. DB and GP were involved in revising the article for important intellectual content. All authors read and approved the final manuscript.
